# Financial Measures to Reduce Carbon Emissions in Britain, Japan and the United States: A SWOT Analysis

**DOI:** 10.3390/ijerph191710771

**Published:** 2022-08-29

**Authors:** Sheng Hu, Pan Zhang, Taoyuan Wei

**Affiliations:** 1 School of Economics, Shandong University of Technology, Zibo 255000, China; 2 CICERO Center for International Climate Research, 0318 Oslo, Norway

**Keywords:** carbon peak, carbon neutrality, financial support, carbon finance, low-carbon society, double carbon goals, carbon emission reduction

## Abstract

To mitigate global warming, China, the world’s largest greenhouse gas emitter, has set the goals of achieving carbon peak by 2030 and carbon neutrality by 2060, and financial measures could play an important role. To avoid unnecessary costs, China could learn from the experience of other countries to better understand the potential role of financial measures in achieving carbon emission reduction goals. Hence, this article adopts a SWOT analysis to compare the financial measures taken by Britain, Japan and the United States in the process of carbon emission reduction in the last twenty years. This article finds that government funds and financial innovation have contributed markedly to carbon emission reduction in those three countries. With the help of the SWOT analysis, we recommend that China take financial measures to help achieve carbon peaking and carbon neutrality goals from four aspects: formulating proper policy, regulating carbon trading market, strengthening international cooperation, and promoting innovation.

## 1. Introduction

The environmental deterioration caused by global warming has had serious impacts on economic activities and society in recent years [1,2]. As greenhouse gas emissions are the main driver of the global warming, increasing numbers of countries have promised to reduce carbon dioxide emissions [3]. For this purpose, as the world’s largest greenhouse gas emitter, China has announced the goal of achieving peak carbon emissions by 2030 and carbon neutrality by 2060 (the “Dual Carbon Goals”) [4]. Financial measures can play an important role in achieving both goals. It has been suggested that China learn from international experience in industrial upgrading, energy adjustment, the development of low-carbon economies [5], and adopting foreign energy and low-carbon technologies to reduce carbon emissions [6]. Few studies have focused on how financial measures could facilitate reducing carbon emissions. Hence, this study will analyze the experience of financial measures adopted in three leading countries, Britain, Japan and the United States, to provide insightful suggestions on low-carbon financial measures for China.

In recent years, China has actively promoted financial participation in carbon emission reduction, developed green finance, and strengthened international financial cooperation to facilitate the realization of the dual carbon goals [7,8,9]. Hence, China’s green finance measures to reduce carbon emissions have developed rapidly, although considerable improvement is necessary compared with the financial measures adopted in certain developed countries such as Britain, Japan and the United States. This motivates us to conduct an overall analysis of the strengths (S), weaknesses (W), opportunities (O) and threats (T) associated with the implementation of financial measures to reduce emissions in the three countries. The SWOT analysis helps us concentrate on the most important factors that are essential for our purpose, although the analysis suffers certain limitations such as ambiguity and lack of mechanism to rank the significance of involved factors.

The analysis is useful for China to improve its existing financial measures to reduce emissions since similar financial measures are probably affected by the same external and internal influencing factors, although certain differences exist between the policy contexts in China and other countries. The novelty of this article is an overall SWOT analysis of the financial measures in Britain, Japan and the United States and what this implies for China to improve its financial measures.

The remainder of the paper is organized as follows. Section 2 briefly reviews related literature. Section 3 presents the materials and the method used in this study. Section 4 briefly introduces the financial measures in the three countries. Section 5 provides an overall SWOT analysis. Section 6 discusses issues related to the analysis including policy implications for China. The last section concludes the paper.

## 2. Literature Review

The common practice of financial measures for carbon emission reduction is to combine low-carbon funds with low-carbon industries, such as increasing financial support for low-carbon technologies and energy [10]. The effectiveness of such measures has been verified in Korea, Russia and other countries [11,12,13,14]. The financial measures are generally promoted by two key partners: governments and financial institutions [15,16,17]. For example, a government can set budget benchmarks and provide financial assistance for carbon-intensive industries (e.g., energy and transportation) to achieve low-carbon development [18,19,20]. Financial institutions can provide low-carbon funds to high-carbon industries through financial innovation, inducing carbon-intensive enterprises to actively participate in emission reduction activities by efficiently using the low-carbon funds [21,22]. While these existing studies have analyzed certain financial measures to reduce emissions taken by both governments and financial institutions, few studies have provided a comparative analysis of the advantages and disadvantages of the financial measures already taken by developed countries, where carbon peak has been achieved and carbon neutrality is targeted for around 2050.

In China, scholars mainly focus on green finance itself from the perspective of financial institutions, rather than financial support for carbon emission reduction [23]. Currently, promoting green transformation has become a development direction pursued by financial institutions in China [24,25], mainly by developing green financial products [26], which are dominated by green funds and green bonds [27,28,29]. Considering the key role of China as the largest global emitter and the potential role of financial measures to reduce emissions, the government and financial institutions in China could adopt more innovative financial measures to reduce emissions by learning from the experiences of other countries, as will be explored by this study.

Green financial instruments can face certain risks, such as the risk of violent fluctuations in financial markets [30]. To effectively prevent these risks, financial institutions themselves can set up risk plans and voluntarily disclose potential risks while government supervision has to be strengthened [31,32]. The government supervision is particularly important for preventing green financial risks since certain risks can be identified earlier and resolved timely [33]. In Japan, the government has established supervision standards through legislation, demanding that financial institutions conducting risk assessment and risk disclosure to strengthen public awareness of the potential risks related to environmental protection [34]. Another risk is related to economic globalization if a domestic green financial market opens to the external market [35] to strengthen international cooperation and promote the globalization of green finance [36]. Hence, a general assessment of risks related to financial measures to reduce emissions would be useful for a country like China.

To sum up, given the existing literature on financial measures to reduce emissions, it is necessary to comparatively analyze the financial measures already adopted by certain developed countries achieving carbon peak. Particularly, an in-depth analysis of the roles of governments and financial institutions would be very useful. The comparative analysis is expected to present the advantages, disadvantages, opportunities and risks related to the financial measures. In doing so, other countries like China could learn from the experience of these pilot countries to achieve carbon peak and, ambitiously, carbon neutrality. Hence, such a comparative analysis will be provided by this study to extend the literature line.

## 3. Materials and Method

### 3.1. Materials

As indicated by the Organization for Economic Cooperation and Development (OECD) statistics [37], Britain is the first country to achieve carbon peak, Japan is the first country to implement energy conservation and environmental protection policies and the United States is the first country to establish a carbon trading platform. In the process of carbon emission reduction, the financial support of these three countries has played a considerable role as described in Section 4. Therefore, Britain, Japan and the United States are selected as research cases, and data and materials are obtained from recent relevant government websites, database and related literature [38,39,40]. Britain’s data are derived from Britain’s government website and related literature [41,42,43,44]; Japan’s data come from the Japanese Ministry of the Environment, the Ministry of Finance and related company announcements [45,46]; and the data for the United States are public data on the carbon market, government documents and related literature [47,48]. For China, data and materials come from the website of the National Bureau of Statistics and related literature [49,50,51]. In addition, carbon emissions data are taken from the OECD website [37].

### 3.2. Analytical Method

This article adopts the SWOT analysis method that permeates the academic peer-reviewed literature for strategic positioning [52]. The method originated in the 1980s and is based on internal and external competitive environments and situational analysis under competitive conditions [53]. Following the method, the factors closely related to the research object, strengths (S), weaknesses (W), opportunities (O) and threats (T), are arranged in a matrix [54,55]. These internal and external factors are systematically and comprehensively analyzed [56,57] to draw clear and concise conclusions. When comparing multiple things, it is easy to show the essential characteristics of different things through a SWOT matrix [58], which can be used for reference by other things through the integration conclusion.

In this study, the practice of financial support for carbon emission reduction in the three countries is presented in the form of a matrix following a SWOT analysis with clear reference to other countries. As far as the method is concerned, SWOT analysis has certain limitations, such as the difficulty determining boundaries between elements and the fact that the analysis can be rigid [59,60]. Therefore, only financial measures are discussed to avoid the problem of dividing the boundaries of elements in this study. Moreover, the different measures of the three countries are dynamically compared in the analysis to avoid the rigidity problem.

In recent years, the SWOT analysis method has been widely used in the financial field to analyze carbon finance [61], green finance [62] and public finance [63]. In this study, data and materials related to carbon emissions and financial measures to reduce carbon emissions of the three chosen countries are collected, analyzed and classified to identify the relationships between financial measures and carbon emission reduction. Then, SWOT analysis is used to analyze and compare the four dimensions (S, W, O and T) of the financial measures for carbon emission reduction in the three countries.

## 4. A Brief Description of Financial Measures in the Three Focused Countries

### 4.1. Britain

Britain achieved carbon peak in 1991 according to the statistics of OECD [37]. Since then, the British government has been committed to reducing carbon emissions. In June 2019, the newly revised Climate Change Act formally confirmed that Britain will achieve carbon neutrality and net zero greenhouse gas emissions by 2050 [64]. The financial measures taken by Britain to reduce carbon emissions can be classified into one of three types: providing governmental funding; establishing carbon funds; and constructing green banks.

#### 4.1.1. Providing Government Funding

The British government and central bank have provided significant funding to reduce carbon emissions. In 2003, the white paper *Our Energy Future: Creating a Low-carbon Economy* announced that carbon emissions by 2050 should be reduced by 60% compared with 2003 through financial investment and other supportive measures [41]. With the support of government finance, Britain has achieved remarkable results in carbon emission reduction [65]. The British government has issued laws to ensure the efficient use of the government funding. In 2008, the British government promulgated the *Climate Change Act* and set up the “carbon budget” decree of the five-year emission plans [42]. Carbon emissions were set 3018 Mt for the period from 2008 to 2012 and 2782 Mt for the period from 2013 to 2017. The carbon budget goals set for both five-year plans have been realized with the support of the government funding, among other measures [66]. The British government specified in the Clean Growth Strategy to increase financial support for CCUS (carbon capture, utilization and storage) technology, and 6 CCUS clusters have been formed [67]. In 2020, the British government also announced a £12 billion investment in the Green Industrial Revolution [68], which is currently underway.

#### 4.1.2. Establishing Carbon Funds

Since 2001, the Britain government has invested in setting up a carbon fund to support private low-carbon efforts. So far, the fund has played an important role in helping enterprises to innovate low-carbon technologies, improve resource efficiency and promote the marketization of low-carbon technologies [69]. In June 2011, the British government invested £560 million to establish a sustainable transport fund to support low-carbon transportation choices, such as public transportation, walking and bicycle sharing [70]. In 2012, local governments provided an additional £40 million to the sustainable transport fund. From 2011 to 2015, the sustainable transport fund provided financial assistance to 96 sustainable transportation projects, which greatly reduced carbon emissions in the field of transportation [71]. In March 2020, Britain announced the CCS Infrastructure Fund (CIF) in the budget for the first time and indicated that the government would invest £1 billion to build CCUS clusters to ensure the goal of zero net emission by 2050 [72].

#### 4.1.3. Constructing Green Banks

The establishment of the Green Investment Bank (GIB) in 2010 is an important measure to provide the necessary funds to achieve the governmental goals of reducing carbon emissions [73]. The GIB has provided increasing green capital investments as follows: GBP 635 million, GBP 668 million, GBP 723 million and GBP 770 million in 2012–2013, 2013–2014, 2014–2015 and 2015–2016, respectively [74]. About 80% of the total funds are used to support five activities: offshore wind power, garbage and recycling, energy efficiency of non-domestic energy, waste to energy and “Green Deal” [75].

### 4.2. Japan

Japan achieved peak carbon emissions in 2013 [37]. Japan has the goal of achieving net zero emission of greenhouse gases by 2050 to fully achieve carbon neutrality [76]. The financial measures taken by Japan to reduce carbon emissions are mainly three types: inducing more green investment by government funding, providing financial benefits to induce social capital into low-carbon projects, and promoting innovation of green finance.

#### 4.2.1. Inducing Green Investment by Government Funding

The Japanese government has invested huge government funding to support low-carbon technology innovation, promote the development of low-carbon industries and to induce more green investment. In 1999, the Ministry of the Environment of Japan set a budget of JPY 86 billion to support sustainable development, including activities to reduce carbon emissions [77]. In 2004, the Development Bank of Japan (DBJ) introduced “promoting environment-friendly operating financing business” to inject money into the field of environmental protection and promote sustainable development [78]. The government has provided financial support for developing new energy-saving, low-carbon and environmentally friendly materials and technologies; promoting the commercialization of low-carbon technologies; and developing green transportation and green energy [76]. An evaluation of Japan’s financial support for environmental projects shows that the emissions in the transportation sector have significantly reduced, and the phenomenon of traffic congestion has been alleviated [79]. It is estimated that the strategy will boost Japan’s economic growth by JPY 90 trillion by 2030 and JPY 190 trillion by 2050 [80].

#### 4.2.2. Providing Financial Benefits to Induce Social Capital into Low-Carbon Projects

The Japanese government encourages social capital to actively participate in carbon emission reduction activities. In the bidding process of the Guangzhisen Solar Power Generation Project in Osaka, Japan [81], the municipal government provided the construction land free of charge, publicly solicited the scheme and invited bids from enterprises for construction. The social responsibility of enterprises was one of the key factors considered in determining the successful bidder. Enterprises in Japan can obtain a tax deduction if they produce energy-saving products or absorb carbon dioxide by other means so that carbon emissions in the production are reduced [46]. Enterprises in low-carbon industries can also receive low-carbon subsidies. In 2020, the Ministry of Economy, Trade and Industry of Japan decided to implement fiscal and taxation benefits and other incentives to mobilize a large amount of private investment into green industries [77].

#### 4.2.3. Promoting Innovation of Green Finance

Japan’s green finance started early and enjoyed rapid innovation. In 2004, the Japanese Ministry of the Environment established the Global Environmental Research Fund to provide financial support for reducing carbon emissions [82]. Thanks to the green fund, Japan achieved carbon peak in 2013 [83]. The New Energy and Industrial Technology Development Organization (NEDO) set up a fund of JPY 2 trillion to support enterprises to implement the latest green and low-carbon technologies for 10 consecutive years [84]. In addition, the Development Bank of Japan issued sustainable development bonds for two consecutive years (2015 and 2016) to promote green development. Many companies have also issued green bonds many times [85]. Japanese banks are the main green financial institutions that promote the low-carbon development of enterprises through preferential interest rates, graded loans and private debt issuance [86]. The extensive use of green financial products has greatly improved the efficiency of Japan’s financial support for carbon emission reduction and accelerated the zero carbon plan process [87].

### 4.3. The United States

In 2007, carbon emissions in the United States achieved the peak [37]. After Biden became President of the United States, he announced the goal of achieving carbon-free power generation by 2035 and striving to achieve carbon neutrality by 2050 [88]. The financial measures taken by the United States to reduce carbon emissions include: providing financial support from the federal and state governments; promoting innovation of financial institutions; and constructing carbon trading market [89].

#### 4.3.1. Providing Financial Support from the Governments

In 1992, the United States promulgated the *Energy Policy Act*, which allowed enterprises and individuals to receive subsidies if they reduced the use of fossil fuel, actively used low-carbon renewable energy and improve energy efficiency [90]. In 2002, the United States promulgated *The Small Business Liability Relief and Brownfield Site Revitalization Act* to reduce the liability of small businesses and set up environmental insurance [91]. These two bills not only reduced carbon emissions but also promoted the development of new energy sources such as wind energy [92].

The federal government has injected significant amounts of money into carbon emission reduction efforts including directly providing financial assistance to related enterprises to reduce carbon emissions. In 2009, the Obama Administration invested $2.4 billion in the automobile industry to actively promote the growth of the electric vehicle industry and reduce the exhaust emissions of traditional fuel vehicles; in 2014, the New York government established the New York Green Bank Project to provide financing for renewable energy projects that are temporarily unable to obtain private investment and to promote green development; From 2016 to 2018, investment in sustainable projects increased from $8.7 trillion to $12 trillion, which resulted in a massive reduction in domestic carbon emission [93]. In 2020, during the United States Presidential election, Biden proposed a plan worth $2 trillion. After being inaugurated as the United States President, he actively promoted this plan. On 19 November 2021, the plan worth $2 trillion was officially passed: The United States government would invest $495 billion to cope with climate change and slow down the rate of climate warming; in addition, the United States has integrated the carbon emission reduction plan with people’s lives. As early as 1936, after the promulgation of the earliest environmental finance bill in the United States, *Act on Bus Exhaust Control*, public transportation was cleaner, and carbon emissions from public transportation were reduced [94].

#### 4.3.2. Promoting Innovation of Financial Institutions

The American capital market is well known for its vastly developed capital market all over the world, which provides strong support for its financial innovation [95]. First, related green bond products have been developed to fill the funding gap of climate change mitigation [96]. Second, financial products such as green insurance and green guarantees have been developed. Green insurance and green guarantees belong to credit transactions; that is, the issuer bears the liability of pollution compensation for enterprises and individual consumers who buy the product. The United States also issued the Green Century Equity Fund and the Pax World Global Green Fund Individual Investor fund to encourage the investment in carbon emission reduction, gas emission, clean energy and other related fields [95]. Third, the banking industry, including state-owned banks and private banks, actively participates in the field of green emission reduction. In 2013, Bank of America proposed to invest $125 billion to support environmental protection business activities by 2025; in 2017, JP Morgan Chase announced that it would invest $200 billion to support green sustainable development before 2025. At present, these two plans are in progress and have achieved good results [97].

#### 4.3.3. Constructing Carbon Trading Market

The United States was the first country to establish a carbon trading platform, with regional trading markets as the main types (i.e., trading markets established within or between states), and the overall scale is limited. In 2003, the Chicago Climate Exchange (CCX), the world’s first standardized climate trading platform and the first institution to implement voluntary participation and legally effective cap and trade, was formally established and put into operation [98]. The main services provided by the CCX are to trade greenhouse gas emission rights and implement membership trading. In addition to emission right trading, the United States carbon trading market also implements a series of incentive measures such as free allocation of carbon allowance and providing certain subsidies for carbon emissions trading projects [99]. The establishment of this trading market provides a reference for other states, and a number of carbon emission reduction projects such as the Regional Greenhouse Gas Initiative (RGGI) and the Western Climate Initiative (WCI) in the United States have been designed and implemented. Different from CCX, RGGI is a mandatory emissions reduction system based on a market model that limits emissions in the power industry and adjusts the plan in time according to historical emissions and operation conditions [100]. WCI is a multinational carbon trading platform that aims to reduce greenhouse gas emission in the involved region. It was jointly established by California in the United States and four Canadian states [101]. The carbon trading market increases the proportion of cleaner production in GDP [102].

## 5. SWOT Analysis of Financial Measures in Britain, Japan and the United States

The above section summarizes the financial measures Britain, Japan and the United States took to reduce carbon emissions. In this section, a SWOT analysis is carried out to analyze the similarities and differences of the financial measures in the three countries. For each country, a SWOT matrix is made to classify the internal and external factors of the financial measures related to strengths, weaknesses, opportunities and threats. Based on the three SWOT matrices, further comparison and analysis are conducted to identify the potential measures to improve for each country. Finally, the similarities and differences of these factors across countries are identified and analyzed.

### 5.1. Britain

#### 5.1.1. Internal Factors

The fact that Britain achieved carbon peak earlier in 1991 [37], meaning much less pressure to cut emissions. Even though, the British government has implemented various financial measures to reduce emissions as introduced above, which are advantages. In terms of disadvantages, the efficiency of the use of government funds is not measured since it is difficult to effectively regulate [103]. In addition, excessive intervention in the financial markets deenergizes the markets and weakens the regulatory functions of the market for carbon reduction. Financial institutions themselves do not participate enough in carbon reduction, lack innovation in their financial products and lack contact with foreign financial markets [71].

#### 5.1.2. External Factors

In terms of opportunities, because of the early carbon peak in the country, the British experience and practice was studied and learned by certain countries to reduce their emissions [64]. Since 1991, the increasing global economic development has facilitated the global flow of finance, which makes it easier to attract foreign funds to invest in carbon reduction. Since Britain’s formal Brexit in 2020, the government autonomy has been greatly improved [104], which may imply more flexible financial measures to reduce carbon emissions.

In terms of threats, British carbon fund-based financial products are mostly used in their own countries amid a lack of international competitiveness [69]. In addition, economic globalization also accelerated the transmission of financial risks, affecting the stability of international financial markets [105]. For security reasons, some transnational financial institutions reduce the flow of funds, making it more difficult to attract foreign investment to reduce carbon emissions [106].

#### 5.1.3. SWOT Matrix and Implications for Improvement

By considering these internal and external factors, we make a SWOT analysis matrix for Britain (Table 1). The matrix highlights certain aspects to be improved for Britain. For example, Combine S and O, Britain is suggested to further develop financial measure to support the realization of clear carbon reduction plans, and introduce foreign funds to support carbon reduction; combining W and O, Britain could improve the efficiency of government funding measures and strengthen low carbon financial innovation; combining S and T, Britain may attract social subjects to participate in carbon emission reduction and prevent financial risks; combining W and T, Britain could actively attract the participation of domestic and foreign financial institutions while maintaining financial market stability.

### 5.2. Japan

#### 5.2.1. Internal Factors

In terms of strengths, Similar to Britain, the Japanese government has implemented considerable financial measures to reduce carbon emissions. Another strength of Japan is the development of green finance, which provides more financial funds for carbon reduction [82,84]. Moreover, Japan’s social participation enthusiasm is high as indicated by the high proportion of social capital for carbon reduction [81].

In terms of weaknesses, Japan’s green finance gives priority to traditional green financial products and pays less attention to new low carbon financial products [107]. Moreover, the efficiency of the use of carbon reduction capital is difficult to measure, which may affect the efficient use of the financial funds from government and social participants [79].

#### 5.2.2. External Factors

In terms of opportunities, thanks to the strong connections between domestic financial institutions and international financial markets, Japan can strengthen financial cooperation and attract foreign funds to support carbon reduction [34]. The strengthening of economic globalization also provides opportunities for Japan to use foreign funds to support carbon reduction. Currently, the continuous expansion of the international green finance market share also helps Japan to strengthen international green finance cooperation [38].

In terms of threats, the existence of Japan’s bilateral credit system makes it face a high exchange rate risk when introducing foreign capital [85]. The influx of a large amount of foreign funds will also have an impact on domestic economic stability. Japan’s domestic financial institutions face different trading rules when they participate in foreign financial markets, which makes the transactions inefficient [81]. When domestic firms are not competitive enough, the lack of attraction to international financial institutions leads to reduced financial support.

#### 5.2.3. SWOT Matrix and Implications for Improvement

A SWOT analysis matrix is made for Japan (Table 2) that shows some improvements in Japan. Combining S and O, Japan could strengthen cooperation with international financial institutions, develop green finance and develop new green financial products; combining W and O, Japan could strengthen financial supervision and improve the efficiency of carbon emission reduction funds; combining S with T, Japan could regulate the participation of social subjects, strengthen the supervision of foreign investment, and maintain economic stability; combining W and T, Japan could develop new financial products, develop energy finance and increase financial support for low-carbon enterprises.

### 5.3. The United States

#### 5.3.1. Internal Factors

One strength of the United States is that the federal and local governments have taken considerable financial measures to reduce carbon emissions. As the global financial center, the United States has developed financial markets, strong financial innovation capability and a variety of low-carbon financial products [95].

In terms of weaknesses, total carbon emissions and per capita emissions in the United States are large [37], which increases the difficulty of financial support for carbon emission reduction. The use efficiency of carbon emission reduction funds is difficult to measure, and the decentralized carbon emission trading market has a negative impact on the United States financial support for carbon emission reduction [102]. In addition, the financial institutions in the country have insufficient awareness of risk prevention, and the financial market is prone to periodic financial crises, which affects the continuity of financial support for carbon emission reduction [108].

#### 5.3.2. External Factors

In terms of opportunities, the United States has more opportunities than Britain and Japan. The position of the global financial center facilitates its enhanced cooperation with international financial markets and facilitates the global issuance of green financial products by the United States, which are purchased by many investors [95]. Every year, many carbon finance professionals in the United States help the United States develop more low-carbon financial products and improve international competitiveness [108].

In terms of threats, the United States has a well-developed financial system but has provided limited low-carbon financial assistance to developing countries [88]. The cyclical financial crisis in the capital market has a great impact on the United States, and the large fluctuations in its currency also increase the risk of low-carbon financial products and reduce their attractiveness to investors [105]. In addition, the different rules of international financial products also increase the issuance cost and transaction cost of green financial products.

#### 5.3.3. SWOT Matrix and Implications for Improvement

Similarly, a SWOT analysis matrix is made for the United States (Table 3) to indicate certain improvement measures for the country. Combining S and O, the United States could strengthen cooperation between government and financial institutions, improve financial innovation and issue more low-carbon financial products for global investment; combining W with O, the country can strengthen the supervision of carbon reduction funds, establish a national carbon trading market, and work with international financial institutions to cultivate carbon finance talents; combining S with T, the United States could increase financial assistance for carbon emission reduction in developing countries, comply with international financial market rules and issue low-carbon financial products, and prevent financial risks; combining W and T, the United States may strengthen capital supervision, improve the efficiency of financial support for carbon emission reduction, and develop more new green financial products to promote carbon emission reduction.

### 5.4. Similarities and Differences Analysis

In this subsection, we compare and analyze the similarities and differences of the three countries’ financial measures for carbon emission reduction. First, regarding the government’s financial support. In all three countries, the government departments have played a leading role in the process of financial support for carbon emission reduction, such as low-carbon technology, low-carbon energy and other products, and announcing policies and regulations regarding the use of the funds to support carbon emission reduction. However, the efficiency of the use of the support funds has not been properly measured. In addition, there are differences between the practices of the three governments. The British government controls more from a macro point of view, pointing out the direction of financial support for carbon reduction. The Japanese government is more cautious about the use of the government budget. The central and local governments of the United States jointly play a role, and local governments have greater autonomy in the use of funds.

Second, regarding the participation of financial institutions, all three countries have developed certain green finance mechanisms (Table 4). Unlike government financial support, financial institutions are more involved in carbon reduction through the provision of green financial products, operating in a cooperative mode between the government and financial institutions. The high degree of financial market liberalization in the three countries makes it convenient for domestic institutions to connect with foreign green financial markets. Domestic financial institutions face many risks in participating in international green financial markets, most notably exchange rate risk [40].

In addition, there are differences in the use of financial products among the three countries. Britain supports carbon reduction by developing carbon funds and establishing green banks. Japan supports carbon reduction mainly through green bonds and green credit. The United States strengthens financial product innovation, constantly updates financial products such as green insurance and green stocks and uses capital advantages to issue green financial products globally.

Third, regarding the participation of social subjects, carbon reduction needs considerable financial support that governments cannot provide alone. Therefore, Britain, Japan, and the United States actively guide social subjects to participate in the construction of low-carbon society. The governments provide financial subsidies and tax reliefs to encourage enterprises to carry out low-carbon transformation and reduce carbon dioxide emissions.

The participation of social subjects in the three countries in carbon emission reduction is also different. The British social subjects have a strong sense of participation in carbon emission reduction. After the government establishes the carbon fund product, the social main body manages and the green bank finally becomes the folk organization. Japanese social subjects mainly participate in the construction of green projects and promote low-carbon innovation. The social subjects of the United States mainly reduce carbon emissions by using low-carbon energy.

## 6. Discussion

### 6.1. Discussion on Analysis Results

As shown in the above SWOT analysis of financial measures for carbon emission reduction, internal and external factors have different impacts. For individual countries, the internal circumstances of each country differ, but the external environment is similar. Therefore, when using financial measures to support carbon reduction, the general conclusion is to give full play to the domestic advantages (S), change the weak conditions (W), seize the opportunity (O) and overcome the threat (T).

In terms of the internal environment, the government and financial institutions play a key role by providing financial funding and actively guiding private participation in carbon reduction financing. The results of the implementation of the measures in the three countries show that direct government financial support has a better and long-term effect on carbon reduction. In contrast, if the government funds are insufficient, support from financial institutions should be given priority. Since most financial institutions pursue profitable businesses, government support in policy, tax relief and other ways is required to encourage financial institutions to support carbon reduction. For any funds to reduce emissions, it is necessary to measure its utilization efficiency indicated by, for example, the project return rate and capital turnover rate. The risks in the process of capital utilization can largely be solved through supervision and the conclusion of default contracts.

In terms of the external environment, countries around the world face similar external factors. Compared with internal factors, external factors tend to have little effect on a country’s domestic financial support for carbon reduction. As shown in the SWOT analysis above, external factors are mainly related to opportunities and threats. Economic globalization and the expansion of global green financial markets have brought opportunities for countries’ financial support for carbon reduction, mainly in the sense of facilitating the free flow of financial capital. More international inflow of funds may reduce the demand for domestic financial support. The international inflow of funds can potentially promote carbon emission reduction activities in countries that have not achieved carbon peak, especially most developing countries. However, note that opportunities and risks coexist. Unrest in international situations can affect the free flow of funds, making it more difficult for countries with less support for carbon reduction to obtain international capital. The existence of international financial risks will also make it prudent to deal with financial capital to reduce carbon emissions. Therefore, it is necessary to strengthen international financial cooperation, improve international financial markets and strengthen financial supervision.

### 6.2. Policy Implications for China

First, the government of China is suggested to make policies and implement financial measures to support carbon emissions reduction. Based on the measures taken by the three countries, the financial support of the government has a significant impact on carbon emission reduction. To reduce carbon emissions, the government can formulate relevant budgets, increase the scale of green investment, and support the development and utilization of CCUS technology [109]. Other measures may include formulating clear and effective plans, setting up relevant policy banks, and guiding the use of funds by the public finance sector. In addition, tax relief and financial subsidies should be provided for carbon emission reduction related industries. Key industries and enterprises that are capable could be supported to take the lead in reaching the carbon peak at a local level.

Second, it will be important to regulate the carbon trading market and improve trading efficiency. It is crucial to improve trading efficiency by promoting the construction of carbon trading market and clarifying the obligations of each trading entity [110]. Compared with Britain, Japan and the United States, China’s carbon trading market is relatively late in construction, although has developed rapidly. Recently a national market has been established in China. To avoid potential cost risks related to the carbon trading market, China can study and learn from the experience of Britain and the United States. Ideally, China could strengthen cooperation with developed countries by jointly formulating the rules of the carbon trading market, promoting the construction of the international market and improving the efficiency of carbon trading.

Third, strengthening international financial cooperation will be significant. In 2008, Industrial Bank Co., Ltd. officially adopted the Equator Guidelines and became the first bank in China to carry out green finance business. Since then, increasing numbers of Chinese banks have launched green finance services, providing large amounts of capital to low-carbon companies and actively participating in international cooperation [111]. Among the Global Certified Climate Bond Issuers in 2021, China Development Bank ranks top one in terms of issuance scale [49]. Even though, the development of financial institutions in China is not perfect in the sense that China can learn the advanced green finance development methods from Britain and Japan to guide financial institutions and other partners to actively participate in carbon reduction.

Fourth, improving financial innovation capabilities and strengthening financial supervision are also critical. Compared with Britain, Japan and the United States, China’s financial innovation capacity is insufficient [112]. China needs to develop green and low-carbon financial products and promote the transformation of green finance. The effectiveness of the financial market transition to a low-carbon economy depends on attracting investors and removing financial market barriers [113]. To attract enterprises to actively reduce carbon emissions, China could innovate more financial products suitable for carbon emission reduction and reduce the financing cost of enterprises. China could also promote its financial innovation by strengthening cooperation with the United States financial institutions and learning advanced technologies.

### 6.3. Limitations

This study has certain limitations. In the analysis method, the analysis accuracy of the SWOT analysis method needs to be improved since the method cannot be used to accurately analyze an issue in complex environments. For this study, the analysis results only apply when the internal and external environments do not change dramatically. Otherwise, it is necessary to adjust the analytical framework and reconsider relevant internal and external factors.

This study selects Britain, Japan and the United States as research cases without taking into account the practices in other countries. This study relies mainly on the data and materials of the past twenty years. However, as many countries have announced carbon neutrality goals, various new financial measures could appear accordingly. Further study may be needed to consider whether there are significant differences between existing and new financial measures to reduce carbon emissions.

## 7. Conclusions

In this study, motivated by what China can learn from other countries, we have analyzed the strengths, weaknesses, opportunities and threats related to financial measures to reduce emissions in Britain, Japan and the United States. The similarities and differences between the three countries are analyzed from three aspects: government’s financial support, participation of financial institutions and participation of social subjects. Policy implications for China are presented from four aspects: proper government policy and financial measures, regulating the carbon trading market, strengthening international financial cooperation and improving financial innovation capabilities.

Further research can be improved from three aspects. First, the SWOT model can be adjusted to consider the changes in the internal and external environments in the future. Second, this study only analyzes general practices in financial support for carbon emission reduction, and thus a comparison between specific practices of financial measures can be conducted to obtain better results. Third, more effective financial measures to reduce emissions might be identified if we compare the past financial measures to reduce emissions with the latest measures that this study has not covered.

## Figures and Tables

**Table 1:** SWOT analysis of financial measures to reduce carbon emissions in Britain.

	Internal factors	Strengths (S)	Weaknesses (W)
External factors		Carbon peak is achieved earlier The government has formulated many incentive policies and invested at a considerable scale every year to reduce carbon emissions Carry out industry-wide carbon reduction activities Green banks play a key role and carbon fund products are fully utilized	No effective supervision of government funds, and the efficiency of fund utilization is not clear Green financial products are mainly carbon funds, and the innovation of low-carbon financial products is slow The government intervenes more in the financial market, and the market lacks vitality Mainly domestic, less contact with foreign financial institutions
Opportunities (O)		SO strategies	WO strategies
The first country to put forward the concept of low-carbon development, and the low-carbon theory has spread abroad. Since 1990s, the global economy has further strengthened Economic independence after leaving the EU has increased		Increase government support and formulate clear carbon emission reduction plans Introduce foreign capital to invest in low-carbon projects Increase financial support for the entire industry and build a low-carbon society Promote the transformation of theoretical achievements and strengthen the innovation of low-carbon financial products	Improve the utilization efficiency of low-carbon funds supported by the government Strengthen links with foreign financial institutions and promote product innovation Reduce direct government intervention in financial markets and increase market vitality Conform to the global economic development trend and develop a low-carbon economy
Threats (T)		ST strategies	WT strategies
Since 1990s, the international society was turbulent and the international financial market was unstable. The ability to innovate financial products is insufficient, and it is difficult to carry out large-scale commercial applications in the international market Less foreign capital enters the country, which has greater financial pressure		Invest capital to innovate low-carbon financial products and put them into the market Guide social capital to join and reduce government financial pressure Reduce external shocks and prevent financial risks	Improve the utilization efficiency of government funds and reduce financial pressure Improve the enthusiasm of financial institutions and design new low-carbon financial products Set the threshold for foreign investment to improve the security of the domestic financial system

**Table 2:** SWOT analysis of financial measures to reduce carbon emissions in Japan.

	Internal factors	Strengths (S)	Weaknesses (W)
External factors		The government invests considerable funds and the speed of low-carbon innovation is faster Green finance is developing rapidly at a large scale Social subjects are highly motivated to participate in carbon emission reduction, and a large amount of social capital is invested in low-carbon projects	The government does not supervise the investment funds, and the efficiency of low-carbon funds is difficult to measure Financial institutions mainly focus on traditional green financial products, with fewer new low-carbon financial products There is no evaluation of the participation of social subjects in green projects
Opportunities (O)		SO strategies	WO strategies
Economic globalization has strenthened and international financial market connections are more convenient The concept of low carbon environmental protection is accepted by most countries Domestic financial institutions are closely linked with international financial institutions The proportion of green finance business of international financial institutions has increased		Conform to the trend of economic globalization and develop green finance Practice the concept of low-carbon environmental protection and invest funds to support low-carbon enterprises Strengthen cooperation with international financial institutions and develop international green finance business Innovate low-carbon financial products and guide social capital investment	Strengthen fund supervision and improve funds use efficiency Guide financial institutions to upgrade their business and develop new green financial services Strengthen international financial cooperation and build international green projects
Threats (T)		ST strategies	WT strategies
The bilateral credit system is greatly affected by exchange rate fluctuations A large number of foreign investments will have an impact on the domestic economy Green financial products have different rules and low transaction efficiency in the global green financial market Insufficient competitiveness will weaken the attractiveness of domestic low-carbon enterprises to international capital		Increase financial support for low-carbon enterprises to improve competitiveness Formulate relevant laws and regulations to regulate the participation of social capital Strengthen capital supervision and prevent financial risks Improve the efficiency of foreign capital utilization and maintain domestic economic stability	Improve the utilization efficiency of domestic and foreign capital and maintain domestic economic stability Design new green financial products to improve the participation efficiency of social subjects Strengthen capital supervision and maintain financial market stability Develop energy finance to help companies make energy transitions

**Table 3:** SWOT analysis of financial measures to reduce carbon emissions in the United States.

	Internal factors	Strengths (S)	Weaknesses (W)
External factors		The capital market is well developed and it is the world financial center Have a high level of financial innovation and advanced low-carbon financial products The federal and local governments jointly invest funds to support carbon emission reduction	The total amount of carbon emissions is relatively large, and the per capita carbon emissions are relatively high The government does not supervise the investment funds, and the efficiency of the use of funds is difficult to measure The carbon trading market is dominated by regional markets, and there is no unified national market Financial institutions have weak awareness of risk prevention, and cyclical economic crises often occur
Opportunities (O)		SO strategies	WO strategies
The scale of the global green finance market continues to expand A large number of carbon finance talents from abroad enter the United States every year It is located in the world financial center and has close ties with the world economy A large number of foreign investors purchase low-carbon financial products in the United States		Increase capital investment and cultivate carbon finance talent Improve the level of innovation and develop more low-carbon financial products Follow the development trend of the global green financial market and issue low-carbon financial products to global investors Strengthen the cooperation between the government and financial institutions to improve the efficiency of capital utilization	Develop more green financial products suitable for carbon emission reduction Strengthen fund supervision and improve fund use efficiency Strengthen cooperation among international financial institutions and cultivate carbon finance talents Establish a national carbon trading market and prevent financial risks
Threats (T)		ST strategies	WT strategies
Provide less low-carbon aid money to other countries, face a crisis of mistrust The cyclical economic crisis has a greater impact on the capital market Dollar fluctuations affect the international competitiveness of domestic low-carbon financial products There are differences in the trading rules of international financial products, increasing transaction costs		Increase low-carbon financial assistance to other countries and enhance the international image Prevent financial risks and maintain the stability of the capital market Strengthen the innovation of low-carbon financial products and improve product competitiveness Comply with international financial product trading rules and reduce transaction costs	Increase government low-carbon funding and provide low-carbon assistance to other countries Strengthen capital supervision and maintain capital market stability Develop new green financial products and improve international competitiveness Build a national carbon trading market and conduct carbon trading with international markets

**Table 4:** The Global Green Finance Development Index and its three component.

Country	Total Score	Policy and Strategy	Product and Market	Global Cooperation	Ranking
Britain	2	1	6	4	2
Japan	6	5	11	1	6
the United States	11	38	4	17	10

Source: IFF 2021 Global Finance and Development Report [38].

## Data Availability

The data used to support the findings of this study are available from the corresponding author upon request.
